# Molecular characterization of *Listeria monocytogenes* isolated from fresh seafood samples in Iran

**DOI:** 10.1186/1746-1596-8-149

**Published:** 2013-09-13

**Authors:** Hassan Momtaz, Shole Yadollahi

**Affiliations:** 1 Department of Microbiology, College of Veterinary Medicine, ShahreKord Branch, Islamic Azad University, ShahreKord, Iran; 2 Post graduated of Master of Science of Microbiology, Falavarjan Branch, Islamic Azad University, Falavarjan, Iran

**Keywords:** Listeria spp, Listeria monocytogenes, Virulence factors, Serotypes, Seafood, Iran

## Abstract

**Background:**

Among all species of *Listeria*, *Listeria monocytogenes* (*L. monocytogenes*) is a major pathogenic microorganism of humans and animals and *L. ivanovii* is rarely pathogenic for humans. The objective of this study was to isolate and characterize *Listeria* species and to determine the frequencies of virulence genes in *L. monocytogenes* serotypes in fresh fish, shrimp, crab and lobster in Isfahan and Shahrekord, Iran.

**Methods:**

From September 2010 to April 2011, a total of 300 marine food samples were purchased from supermarkets of Isfahan and Shahrekord cities, Iran. All samples were cultured and the positive samples for *L. monocytogenes* were analyzed for presence of serotypes and virulence genes.

**Results:**

From the total 300 samples, 23 (10.45%) fresh fish and 1 (2.5%) shrimp samples were positive for *Listeria* spp., but there were no positive lobster and crab samples for *Listeria* species. Only *L. monocytogenes* was isolated from 17 fish (7.25%) and 1 shrimp (2.5%) samples while *L. innocua*, *L. ivanovii* and *L. seeligeri* only detected in fish samples (2 (0.9%), 3 (1.36%) and 1 (0.45%)), respectively. The *plcA, prfA, actA, hlyA* and *iap* virulence genes were detected in all of the 18 *L. monocytogenes* isolates. Totally, the 4b, 1/2a and 1/2b serotypes were detected in 66.66%, 5.55% and 27.77% bacterial isolates, respectively.

**Conclusions:**

Consumption of these sea foods, either raw or undercooked, may contribute to food-borne illness due to *L. monocytogenes* in Iran. The hygienic quality of sea food products should be observe.

**Virtual slides:**

The virtual slide(s) for this article can be found here: http://www.diagnosticpathology.diagnomx.eu/vs/3422944359800606

## Background

Some-food borne diseases are well recognized but their prevalence rate has been increased these days. Totally, *Campylobacter* species (*Campylobacter* spp.), *Salmonella* spp., *Listeria* species (*Listeria* spp.), and *Escherichia coli* O157:H7 have been generally found to be responsible for majority of food- borne outbreaks [1,2]. *Listeria* spp. are ubiquitous, Gram-positive, facultative anaerobic, non-spore-forming, rod-shaped bacteria. *Listeria monocytogenes* (*L. monocytogenes*), *L. ivanovii*, *L. innocua*, *L. seeligeri*, *L.welshimeri*, and *L. grayi* are the most important species in the genus *Listeria*[3].

The hemolytic species of *Listeria* such as *L. monocytogenes*, *L. ivanovii* and *L. seeligeri*, are associated with human pathogenicity. Both *L. ivanovii* and *L. seeligeri* are the rare causes of human infection. These two pathogens are responsible for meningitis in a nonimmunocompromised adult [4]. The *L. monocytogenes* has been involved in known food-borne outbreaks of listeriosis [5,6] however, there are some reports of *L. seeligeri* and *L. ivanovii* causing food-borne illness in humans [7,8]. The *L. monocytogenes* is responsible for listeriosis which can known by several complications including abortion, bacteraemia, sepsis, and meningoencephalitis [9,10]. The ability of *L. monocytogenes* to survive in a wide range of environmental conditions like grow at refrigerator temperatures is undeniable.

Approximately, 2,500 cases of human listeriosis occur annually in the United States, resulting in 500 deaths [11]. Since, *Listeria* spp. has been isolated from a wide range of sea-food products such as shrimp [12], crab [13], cold-smoked rainbow trout [14], fish products [15] and lobster [16]. Multiple virulence factors such as hemolysin (hlyA), phosphatidylinositol phospholipase C (plcA), actin polymerization protein (actA) and invasive associated protein (iap) are important in the pathogenesis of *L. monocytogenes* infections [17]. The 1/2a, 1/2b, and 4b are the most commonly detected serotypes of *Listeria* spp. isolated from various types of clinical samples [18]. Isolation and characterization of *Listeria* species is done mainly by cultural, biochemical and molecular methods. There is a need for quick and reliable molecular methods such as the Polymerase Chain Reaction (PCR) for *Listeria* detection.

Sea-foods are so popular among Iranian people. The epidemiology and prevalence of *Listeria* spp. is essentially unknown in Iranian sea-foods. Therefore, the present study was carried out in order to detection and characterization of *Listeria* spp. and study the frequency of virulence genes and serotypes of *L. monocytogenes* isolated from fresh fish, crab, shrimp and lobster using cultural, biochemical and molecular techniques.

## Methods

### Bacterial strains

The standard strains of *L. monocytogenes* (PTCC 1298), *Listeria ivanovii* subsp. *ivanovii* (PTCC 1303), *Staphylococcus aureus* (PTCC 1113) and *Rhodococcus equi* (PTCC 1633) were obtained from the Iranian Research Organization for Science and Technology, Iran and used in culture and PCR methods.

### Sample collection

From September 2010 to April 2011, a total of 300 seafood samples including fresh fish (n = 120), crab (n = 20), lobster (n = 40) and shrimp (n = 120) were collected from the supermarkets and retailers of Isfahan and Shahrekord cities, Iran. Samples were transferred to the Food Microbiology Laboratory at the Islamic Azad University of Shahrekord Branch in portable insulated cold-boxes. Samples were analyzed on a day of collection.

### Isolation and Identification of Listeria

*Listeria* spp. were isolated from seafood samples according to ISO 11290 protocol [19]. Then all isolates were subjected to standard biochemical tests including Gram staining, catalase test, motility test at 25°C and 37°C, acid production from glucose, manitol, rhamnose, zylose, α -methyl-D-mamoside, and nitrate reduction, hydrolysis of esculin, MR/VP test, ß-hemolytic activity, and CAMP test [20].

### Phenotypic characterization

1- Haemolysis on sheep blood agar (SBA)

All the *Listeria* isolates were tested for the type (α or β) and the degree (narrow or wider) of hemolysis on 7% sheep blood agar (SBA). Briefly, the isolates were streaked onto 7% SBA plates and incubated at 37°C in a humidified chamber for 24 h and examined for hemolytic zones around the colonies. Interpretation of the hemolytic reaction was based on the characteristic β-hemolysis in the form of wider and clear zone of hemolysis representing *L. ivanovii* while a narrow zone of α-hemolysis was the characteristic of *L. monocytogenes* or *L. seeligeri*[21].

2- Christie, Atkins, Munch and Petersen (CAMP) test

All the *Listeria* isolates were tested by CAMP test. Briefly, the standard strains of *Staphylococcus aureus* and *Rhodococcus equi* were grown overnight on 7% SBA plates at 37°C and their colonies were again streaked onto freshly prepared 7% SBA plates in a manner such that the streaks were wide apart and parallel to each other. In between the parallel streaks of *S. aureus* and *R. equi* the *Listeria* isolates were streaked at 90°C angle and 3 mm apart before incubating them at 37°C for 24 h. The plates were examined for enhancement of the hemolytic zone from partial hemolysis to a wider zone of complete hemolysis, if any, between a *Listeria* strain and the S. aureus or R. equi strain owing to the synergistic effect of their hemolysins in case of a CAMP-positive reaction. The *Listeria* isolates with CAMP-positivity against *S. aureus* were characterized as *L. monocytogenes* and those with CAMP positivity against *R. equi* were characterized as *L. ivanovii*[21].

3- Phosphatidyiinositol- specific phospholipase C (PI-PLC) assay

All the biochemically characterized *Listeria* isolates were assayed for PI-PLC activity as per the method of Leclercq [22] with certain modifications. In brief, the *Listeria* isolates were grown overnight onto 7% SBA plates at 37°C. All *Listeria* isolates were streaked on *L.* mono differential agar (Hi Media Ltd, Mumbai, India) in order to assess PI-PLC activity. The inoculated plates were incubated at 37°C in a humidified chamber for 24 h. On L. mono differential agar, light blue colonies showing a halo formation around the inoculation site were considered positive for PI-PLC assay.

4- Phosphatidyiinositol- specific phospholipase C (PC-PLC) assay

The eggyolk opacity test was done to examine the phosphatidylcholine-specific phospholipase C (PCPLC) activity of the isolates. Tryptic soy agar (Hi Media Ltd. Mumbai, India) plates were prepared with 2.5 per cent egg-yolk emulsion (Hi Media Ltd. Mumbai, India) and 2.5 per cent NaCl, pH 6.5-7. *Listeria* isolates were streaked onto the agar surfaces and incubated at 37°C for 36–72 h and observed for formation of opaque zones surrounding the growth [23].

### DNA extraction

Chromosomal DNA was prepared using the Zhang et al. [24] method. Briefly, 1 mL of overnight culture (from brain–heart infusion) broth was transferred to 1.5-mL microfuge tube and centrifuged at 8,000 rpm for 5 min, and the supernatant was discarded and 500 μL of cetyl trimethylammonium bromide buffer at 60°C was added to the microfuge tube containing the bacterial pellet; the mixture was held in water bath at 64°C for 20 min. During incubation, the mixture was briefly mixed several times. After incubation, 500 μL of chloroform/octanol (24:1) was added and mixed vigorously followed by centrifugation at 3,000 rpm for 5 min. The supernatant was transferred to a clean microfuge tube, and an equal volume of ice-cold isopropanol was added and kept on ice bath for 2-h precipitation. The solution was then centrifuged at 8,000 rpm for 8 min. The aqueous phase was discarded and the DNA pellet was rinsed with 80% ethanol, air- dried and resuspended in 50 μL of double distilled water and used for PCR [25].

### PCR condition for detection of Listeria spp. L. monocytogenes serotypes and virulence genes of L. monocytogenes

The details of the primers sequences for amplification of *Listeria* spp., *L. monocytogenes*, its virulence genes and serotypes are shown in Table 1. DNA amplification was performed in a DNA thermal cycler (Eppendrof Mastercycler 5330; Eppendorf-Nethel-Hinz GmbH, Hamburg, Germany). The PCR programs and their volumes for *L. monocytogenes*, *L. innocua*, *L. ivanovii, L. seeligeri, L. welshimeri,* and *L. grayi* amplification were studied using the method which was described by Bubert et al. [26].

**Table 1:** **Primers for amplification of virulence associated genes, *Listeria *spp. and serotypes of *L. monocytogenes***

**Primer name**	**Primer sequence (5′-3′)**	**Target**	**Size of product (bp)**	**References**
**Lis1B**	TTATACGCGACCGAAGCCAAC	*L. innocua*	870	[26]
**Ino2**	ACTAGCACTCCAGTTGTTAAAC			
**Lis1B**	TTATACGCGACCGAAGCCAAC	*L.*	660	[26]
**MonoA**	CAAACTGCTAACACAGCTACT	*monocytogenes*		
**Lis1B**	TTATACGCGACCGAAGCCAAC	*L. ivanovii*	1100	[26]
**Iva1**	CTACTCAAGCGCAAGCGGCAC			
**Lis1B**	TTATACGCGACCGAAGCCAAC	*L. seeligeri*	1100	[26]
**Sel1**	TACACAAGCGGCTCCTGCTCAAC			
**Lis1B**	TTATACGCGACCGAAGCCAAC	*L. welshimeri*	1050	[26]
**Wel1**	CCCTACTGCTCCAAAAGCAGCG			
**Lis1B**	TTATACGCGACCGAAGCCAAC	*L. grayi*	480	[26]
**MuraI**	GTGATTTCTGCTTGCCATAG			
**prsF**	GCTGAAGAGATTGCGAAAGAAG	All *L. monocytogenes* serovares	370	[27]
**prsR**	CAAAGAAACCTTGGATTTGCGG			
**lmo0737F**	AGGGCTTCAAGGACTTACCC	*L. monocytogenes* serovar1/2a	691	[27]
**lmo0737R**	ACGATTTCTGCTTGCCATTC			
**ORF2819F**	AGCAAAATGCCAAAACTCGT	*L. monocytogenes* serovar1/2b	471	[27]
**ORF2819R**	CATCACTAAAGCCTCCCATTG			
**ORF2110F**	AGTGGACAATTGATTGGTGAA	*L. monocytogenes* serovar 4b	597	[27]
**ORF2110R**	CATCCATCCCTTACTTTGGAC			
**plc A-F**	CTGCTTGAGCGTTCATGTCTCCATCCCCC	*plcA* gene	1484	[28]
**plc A-R**	CATGGGTTTCACTCTCCTTCTAC			
**prf A-F**	CTGTTGGAGCTCTTCTTGGTGAAGCAATCG	*prfA* gene	1060	[28]
**prf A-R**	AGCAACCTCGGTACCATATACTAACTC			
**act A-F**	CGCCGCGGAAATTAAAAAAAGA	*actA* gene	839	[29]
**act A-R**	ACGAAGGAACCGGGCTGCTAG			
**hly A-F**	GCAGTTGCAAGCGCTTGGAGTGAA	*hlyA* gene	456	[30]
**hly A-R**	GCAACGTATCCTCCAGAGTGATCG			
**Iap-F**	ACAAGCTGCACCTGTTGCAG	*Iap* gene	131	[31]
**Iap-R**	TGACAGCGTGTGTAGTAGCA			

The PCR was standardized for the detection of virulence associated genes of *L. monocytogenes* by previously described methods [26-31].

The multiplex PCR assay was standardized for the detection of three major serovars of *L. monocytogenes* namely 1/2a, 1/2b and 4b, using the method which was described by Doumith et al. [27]. The PCR products were analyzed by 1.5% agarose gel electrophoresis and the specific DNA bands were visualized using ethidium bromide staining under UV illumination.

## Results and discussion

All of the three houndred sea-food samples were studied for presence of *Listeria* spp. Totally, twenty three fresh fish samples (10.45%) and only one shrimp sample (2.5%) were positive for *Listeria* spp. there were no positive results for lobster and crab samples. Also, 17 fresh fish samples (7.72%) were positive for the *L. monocytogenes*. Totally, the frequency of *L. innocua*, *L. ivanovii* and *L. seelgeri* in fresh fish samples of our study were 0.9%, 1.36% and 0.45%, respectively (Table 2). One of the shrimp samples of our study was positive for *L. monocytogenes* (2.5%).

**Table 2:** **Prevalence of *Listeria *spp. in marine foods in Iran**

**Type of sample**	**No. of samples**	**No. (%) of *Listeria *spp.**	**No. (%) of *L. monocytogenes***	**No. (%) of *L. innocua***	**No. (%) of *L. ivanovii***	**No. (%) of *L. seeligeri***
**Fish**	220	23 (10.45)	17 (7.72)	2 (0.9)	3 (1.36)	1 (0.45)
**Shrimp**	40	1 (2.5)	1 (2.5)	-	-	-
**Lobster**	20	-	-	-	-	-
**Crab**	20	-	-	-	-	-
**Total**	300	24 (8)	18 (6)	2 (0.66)	3 (1)	1 (0.33)

All the 18 isolates of *L. monocytogenes* showed the characteristic enhancement of hemolytic zone with *S. aureus*. Also, all of the 18 isolates of *L. monocytogenes* were found to be pathogenic by PI- PLC and PC-PLC.

The five virulence-associated genes (*plcA, prfA, actA, hlyA* and *iap*) were detected in all of the 18 *L. monocytogenes* isolates. The most commonly detected serotype in *L. monocytogenes* isolates was 4b, which occurred in 12/18 (66.66%) samples. The frequency of 1/2a and 1/2b serotypes were 5.55% and 27.77%, respectively (Table 3).

**Table 3:** **Frequency of *Listeria monocytogenes serotypes *in marine foods in Iran**

**Type of sample**	**No. (%) of *L. monocytogenes***	**4b**	**1/2a**	**1/2b**
**Fish**	17	11 (64.70%)	1 (5.88%)	5 (29.41%)
**Shrimp**	1	1 (100%)	-	-
**Total**	18	12 (66.66%)	1 (5.55%)	5 (27.77%)

Listeriosis is one of the most important zoonotic bacterial diseases with worldwide distribution. Disease has considerable economic and public health importance.

*Listeria monocytogenes* has been described as opportunistic pathogen mainly affecting children, pregnant women, and aged and immune-challenged individuals [32,33]. Also, a wide variety of animals including sheep, cattle, goats, pigs, rabbits, mice, birds, and fish are also infected with *L. monocytogenes*. An atypical foodborne listeriosis has a range of 25 to 30% in susceptible populations [34]. Since 1975, foodborne listeriosis outbreaks have been reported in industrialized countries of Europe, North America and Oceania with few or no reports from Africa, Asia and Latin America [35,36]. Despite the high importance of sea-foods listeriosis, there were few published data about its distribution in fish and shrimp samples of Iran [12,37,38].

Our results revealed that 6%, 1%, 0.66% and 0.66% of Iranian sea-food samples were positive for *L. monocytogenes*, *L. ivanovii, L. innocua* and *seeligeri*, respectively. Rahimi et al. (2012) reported that *L. monocytogenes* and *L. innocua* were detected in 1.9% and 5.7% of the frozen and fresh sea-food samples, respectively [12]. Zarei et al. [38] reported the low frequency of *L. monocytogenes* in Iranian sea-food samples (1.4%). Also, Akhondzadeh Basti et al. [37] reported that 2.6% of smoked fish samples were positive for *L. monocytogenes*. Study in Urumia, Iran showed that 12.37% of collected fish samples were positive for *Listeria*[39]. They showed that 21% and 29% of isolates were *L. monocytogenes* and *L. ivonoi*[39]. The results of our study and several Iranian reports showed that the *Listeria* spp. had the low frequency in Iranian sea-foods.

An overall prevalence of *L. monocytogenes* was 3% in European fish [40] but Miettinen and Wirtanen [41] reported that the prevalence of *Listeria* spp. and *L. monocytogenes* in pooled unprocessed fresh rainbow trout were 35% and 14.6%, respectively. The *L. innocua* was the most common *Listeria* spp. in the fishes of Greece country [42]. The incidence of *Listeria* spp. in Turkey was 30% in freshwater samples and 10.4% in marine fish samples. Also, 44.5% and 83.5% of all isolates were *L. monocytogenes* and *L. murrayi*, respectively [43]. These high differences in prevalence of *Listeria* spp. in sea-foods maybe due the facts that type of samples (fish, shrimp, crab, oyster and lobster), number of samples, methods of sampling, method of experiment, geographical area and even climate of area which samples were collected are different in each investigation.

All of the detected *L. monocytogenes* bacteria had *plcA, prfA, actA, hlyA* and *iap* putative virulence genes. Unfortunately, there were no previously published data about detection of *L. monocytogenes* virulence factors in sea-food products but their high frequencies in the bacterial strains of our study can lead to adhesion, invasion and epithelial damage to the human digestive system.

The most commonly detected serotypes in *L. monocytogenes* isolates our study was 4b (66.66%), followed by 1/2b (27.77%) and 1/2a (5.55%). Similar results have been reported previously [26,27,44]. Previous report of the National Reference Center in France showed that over than 98% of 5,000 isolates of *L. monocytogenes* harbored 1/2a, 1/2b, 1 /2c, and 4b serotypes [26].

## Conclusions

The results of our study showed that severe controls should be performed on the hygienic quality of Iranian sea-foods. These products are well contaminated with *Listeria* spp. and especially *L. monocytogenes*. Contact with intestinal contents, cross contamination from infected staffs, using contaminated equipments, fish manipulation and inappropriate transportation are the main factors for sea-foods contamination. Also, may be some food safety and quality standards need to be applied and performed in most of Iranian supermarkets and even fishing centers to control growth of *Listeria* during fishing, collection, transmission, distribution and storage periods. Suitable cocking of sea-foods can diminish the microbial loads of these products especially for *Listeria* spp.

## Competing interests

Both authors declare that they have no competing interests.

## Authors’ contributions

The DNA extraction, PCR techniques, statistical analysis, writing of manuscript and supporting of project was performed by HM and samples collection and culture was performed by SY. Both authors read and approved the final manuscript.
